# A Free Service and Purchasing Agency

**Published:** 1914-03-21

**Authors:** 


**Please detach this section and carefully preserve it.**

### THE DOCTOR'S WIFE

### THE DOCTORS' WIVES ASSOCIATION.

### A FREE SERVICE AND PURCHASING AGENCY.

BY A DOCTOR'S WIFE.

These words, "The Doctor's Wife," call up many different pictures in many different minds, but to those who really know, the picture is, in the majority of cases, one of great beauty and worthy of sincere respect.

The doctor's wife must be all things to one man, otherwise she is not a success in her chosen path of life. In addition to being his wife she must be his partner, his assistant, and very often she has been his dispenser, also his housekeeper and sometimes his financial adviser, as well as administrator of his income. Considering these unending claims upon her time, thought, and energy, it is with great satisfaction that one turns to a well-thought-out plan by means of which this most valuable citizen may enjoy a measure of compensation for her more or less self-sacrificing life. This plan, which has been built up for the sole purpose of benefiting the doctor's wife, is one which carries with it one imperative condition, and one only—viz. the loyal and solid union of all those concerned.

I think the majority of people will admit that, however large their income or allowance, it is never quite large enough; and in the case of the wife of a hard-working general practitioner this fact is always in the foreground. If one could present a scheme which would cut down expenses at the rate of 10 to 15 per cent., I think such a scheme would receive a royal welcome. Only those who know from hard experience what it means to make an allowance for one person do duty for three, will be able to appreciate the delight of having "something over" to spend as one inclines, even to be extravagant if one so chooses. Only the woman who has to buy clothes, not because of their beauty or grace, but because of their enduring qualities, can experience the real joy of securing a Paris model. We have spoken of clothes in connection with this scheme for reducing prices, but the same holds good in other equally important matters with the exception of food—viz., the buying and replenishing of household goods and the whole general upkeep of the house; the question of holidays, and the education of the children; all these matters can, and will, be included in this scheme for the general good of the doctor's wife.

This Association which we propose to help the doctor's wife to form will have as its members the wives of doctors who subscribe to The Hospital. There will be membership by subscription. and all that is necessary is that the doctor or the doctor's wife shall take The Hospital for one year. As a result the ladies will be able to obtain the best of everything at a lower cost than could be secured in any other way. The scheme is so complete and so far-reaching that, with the exception of foodstuffs, there are few things the doctor's wife may not have at a much lower price than is paid by the general public. With regard to holidays, not only can these be enjoyed at a moderate cost, but every kind of holiday can be arranged, whether at home or abroad, by land or sea, for a short or a long period. Expert advice will be at the service of the doctor's wife, and if she desires a trip to the Continent or a visit to some country which is unknown to her, our expert officials can very quickly draw up a detailed programme complete in every respect. Our client can then go off with a clear mind, feeling absolutely free, not only to enjoy every moment of the well-earned holiday, but free also to absorb all the health-giving rest to which she is entitled, unmarred by any anxiety as to what is the next thing to be done, and the best way to go about it. A man who has spent himself so thoroughly in the service of humanity has no desire to work hard at holiday-making; he finds it much better to yield himself up to the care of the expert, since he knows he is in good hands, and that all will be arranged and carried out in the best possible manner.

To turn to another branch of the work of the Association—if a doctor is going to be married, his bride can be certain of furnishing her new home in whatever style she chooses at a much reduced cost; at the same time she can avail herself of expert advice upon every detail of the whole establishment, as painting and papering, the hot-water system, the plumbing, and many other subjects of which a young wife can have little knowledge or experience. She very naturally desires to make her home as nearly perfect as she can, but a limited income is the arresting voice to which she must listen, and it is not to be wondered at that she looks upon the forbidden things as the most desirable, so that their value increases by leaps and bounds because they appear to be quite beyond her reach. It is to some extent a fictitious value with which she endows them, and this new scheme will help her to prove it. There are to-day many labour-saving devices on the market which, if one could possess, would make life, especially in large towns, much easier and more satisfying, but when one begins to count up and consider the sum of money necessary to possess these it rapidly assumes alarming dimensions. At this point, however, the Association offers everything that is desired at a considerably lower price, and will also be ready to give expert advice upon kind and quantity, as well as upon the amount of money it would be right to spend upon the different items of household management, taking into consideration the whole income and all legitimate claims upon it. It is a serious undertaking for a young, inexperienced wife to spend a relatively large sum of money in fitting up a house, especially as the money is often the result of hard, enduring work and much self-denial on the part of the young husband. Any woman who loves her husband and her home will look upon the disposal of the money as a very sacred trust, and one not to be lightly entered upon. She cannot herself acquire the experience necessary for the best and wisest expenditure, but, if she joins the Doctors' Wives Association, she can secure reliable advice from experienced buyers, whose business it is to do the very best for the members. If 1,000 such women sign and send in the coupon on p. 678 a new era will open for the doctor's wife.

